# Comparison of Ultrasound-Guided Versus Palpatory Method of Posterior Tibial Artery Cannulation: A Prospective Randomized Controlled Study

**DOI:** 10.7759/cureus.51087

**Published:** 2023-12-25

**Authors:** Jhansi Eda, Priyanka Gupta, Ashutosh Kaushal

**Affiliations:** 1 Anaesthesiology, All India Institute of Medical Sciences, Rishikesh, Rishikesh, IND; 2 Anesthesiology and Critical Care, All India Institute of Medical Sciences, Rishikesh, Rishikesh, IND; 3 Anaesthesiology, All India Institute of Medical Sciences, Bhopal, Bhopal, IND

**Keywords:** cannulation attempts, first attempt success rate, arterial cannulation, palpatory technique, ultrasound, posterior tibial artery

## Abstract

Introduction: Superficial arteries, such as radial and dorsalis pedis arteries, are commonly cannulated for invasive blood pressure monitoring. Failure to cannulate these arteries necessitates alternate arteries, such as the posterior tibial artery (PTA). The deep-seated anatomy of PTA makes arterial cannulation precarious by the palpatory technique. Ultrasound guidance during PTA cannulation may overcome this problem. With this background, we evaluated the ultrasound-guided (USG) versus palpatory method for PTA cannulation with respect to the first attempt's success and number of attempts.

Methods: A total of 240 American Society of Anesthesiology (ASA) physical status I-IV adult patients undergoing major surgeries requiring arterial cannulation were randomly allocated (1:1) to group A (USG-guided cannulation, n = 120) and Group B (cannulation by palpatory technique, n =120). PTA was cannulated by either of the techniques according to randomization. Data were analyzed and compared in both groups for first-attempt success, number of attempts, assessment time, cannulation time, and complications.

Result: The successful cannulation in the first attempt in Group A was 25.8% (n = 31), and in Group B, it was 12.5% (n = 15) (p = 0.009). In Group A, 78.3% of patients (n = 94) had successful cannulation, and in group B, 65% of patients (n =78) had successful cannulation (p = 0.022). Both groups had similar assessment time (p = 0.348) and cannulation time (p = 0.864).

Conclusion: USG-guided PTA cannulation offers a greater chance of success without any added increase in procedure time.

## Introduction

Intra-arterial cannulation is a commonly used method for continuous beat-to-beat blood pressure monitoring, blood sampling, and arterial blood gas analysis during the intraoperative period in major surgeries and in intensive care units. The radial artery is the preferred site for cannulation because of its superficial course, collateral supply by the ulnar artery, and lower risk of complication [1]. In certain circumstances, such as failure of radial artery cannulation, patients with upper limb trauma, edema, burns, or surgeries on the arms and forearms, and patients with carpal tunnel syndrome, other arteries are assessed for cannulation. The other commonly assessed arteries for cannulation include the femoral artery, dorsalis pedis artery, and posterior tibial artery (PTA) [2]. The feasibility of dorsalis pedis artery cannulation as an alternative to radial artery cannulation has been studied previously [3]. There is a paucity of literature regarding post tibial artery cannulation. The PTA is a branch of the popliteal artery. It is deeper and has a larger diameter than the radial artery. The PTA also has extensive collateral circulation; thus, its cannulation lowers the risk of distal ischemia. It has been shown that cannulation of the PTA is comparable to radial artery cannulation and even better than dorsalis pedis artery cannulation in children of <2 years of age [4]. The traditional method for arterial cannulation is the landmark technique of palpating the arterial pulse. However, ultrasound guidance facilitates arterial catheterizations, especially in certain situations like shock, hypotension, obesity, coagulopathy, and failure of the palpatory technique of arterial catheterization [5]. Very few studies are available comparing the palpatory method with ultrasound-guided (USG) PTA cannulation in adults. As the PTA is deep-seated, we hypothesized that USG PTA cannulation would be better than conventional palpatory technique concerning first-attempt success rates and reducing the number of attempts.

## Materials and methods

This prospective randomized study was done in one of the tertiary hospitals in India. The Institutional Ethics Committee of All India Institute of Medical Sciences, Rishikesh, approved the study protocol (313/IEC/PGM/202020/06/2020). Before patient enrollment, the study protocol was registered with the clinical trial registry of India (CTRI/2020/08/027199, 18/08/2020). The study was conducted in accordance with the Declaration of Helsinki. Written informed consent was obtained from all the participants.

All American Society of Anaesthesiologists (ASA) status I-IV patients, aged 18-65 years of either gender, undergoing major elective or emergency surgery requiring arterial cannulation, were included in the study. Patients who refused consent, with absent arterial pulsations (following anesthesia induction), with skin erosions near the cannulation site, patients having prevalent arteriosclerotic changes, and patients with Raynaud’s or peripheral vascular disease were excluded. Morbidly obese patients, patients in shock, and patients undergoing surgery on lower limbs were also excluded.

Patients were allocated after randomization into two parallel groups (allocation ratio 1:1) by computer-generated random tables (using Research Randomizer, Social Psychology Network, http://www.randomizer.org). The allocation was done with the help of a serially numbered opaque sealed envelope. The studied groups were Group A, which used the USG technique, and Group B, which used the conventional palpation technique.

Once the patient was shifted to the operating room, standard monitors were applied. The patients were induced with general anesthesia using a standard protocol. With all aseptic precautions, a 20G arterial cannula (BD Arterial Cannula, Becton Dickinson Infusion Therapy Systems Inc., USA) was used to percutaneously puncture the posterior tibial artery (PTA) either by the palpation technique in the groove between the medial malleolus and tendon of Achilles or by the USG (GE Health Care co, Tokyo Japan) technique using a linear probe transducer (6-12 MHz) and real-time visualization of the artery.

For the US technique, limb position was made by ankle dorsiflexion up to 90 degrees and eversion of the foot. The PTA was viewed first in a short-axis view, and then the transducer was rotated to 90 degrees, keeping the image in the center of the US screen to identify the artery in its long axis. The needle was inserted by the in-plane technique. All the cannulations were performed by a single trained investigator to minimize inter‑individual bias. The investigator was trained for PTA cannulation for both palpatory and USG techniques and performed at least 50 cannulations using both techniques before the start of this study.

Only one side of the PTA was attempted during the study, and the following data were collected. The assessment time defined as "time taken from the start of palpation/screening of the artery to just before the skin puncture by needle," and the cannulation time was defined as “time taken from the initial skin penetration by the needle till successful cannulation of arteries.” First attempt success was described as “successful cannulation following first-time skin puncture.” Successful arterial cannulation was defined as “removal of the stylet and successful threading of cannula into the artery.” The number of attempts was defined as “the number of times skin is freshly punctured with the needle.” The success rate was defined as “successful cannulation of the PTA in ≤3 attempts” on the same side.

The primary outcome of the study was the successful cannulation at the first attempt. The secondary outcomes were assessment time, cannulation time, number of attempts, and cannulation outcome. Any complications, such as arterial spasm, thrombosis, or tissue necrosis, were noted. Any untoward events after successful cannulation were also noted, such as absence of backflow after fixing the cannula or after positioning of the patient for surgery. 

Sample size calculation was based on previous studies comparing the radial artery with the PTA [4].^ ^To compare the 75% first-attempt success rate in the USG technique with the palpatory group, with 95% power and 0.05 two-sided type I error rate, we required the enrollment of 240 patients.

Data were coded and recorded in the MS Excel spreadsheet program (Microsoft, USA). IBM SPSS Statistics for Windows, version 23 (released 2015; IBM Corp., Armonk, New York, United States) was used for data analysis. Descriptive statistics were elaborated in mean ± standard deviations (SDs) and medians-interquartile ranges (IQRs) for continuous variables and frequencies and percentages for categorical variables. Group comparisons for continuously distributed data were made using an independent sample t-test (two groups) and one-way analysis of variance (ANOVA) (more than two groups). Post-hoc pairwise analysis was performed using Tukey’s honestly significant difference (HSD) test in the case of one-way ANOVA. For non-normally distributed data, appropriate non-parametric tests in the form of the Wilcoxon test/Kruskal-Wallis test were used. The chi-squared test was used for group comparisons of categorical data. Fisher's exact test was used instead if the expected frequency in the contingency tables was <5 for >25% of the cells. Linear correlation between two continuous variables was explored using Pearson’s correlation (normally distributed data) and Spearman’s correlation (non-normally distributed data). Statistical significance was kept at p < 0.05.

## Results

**Figure 1:**
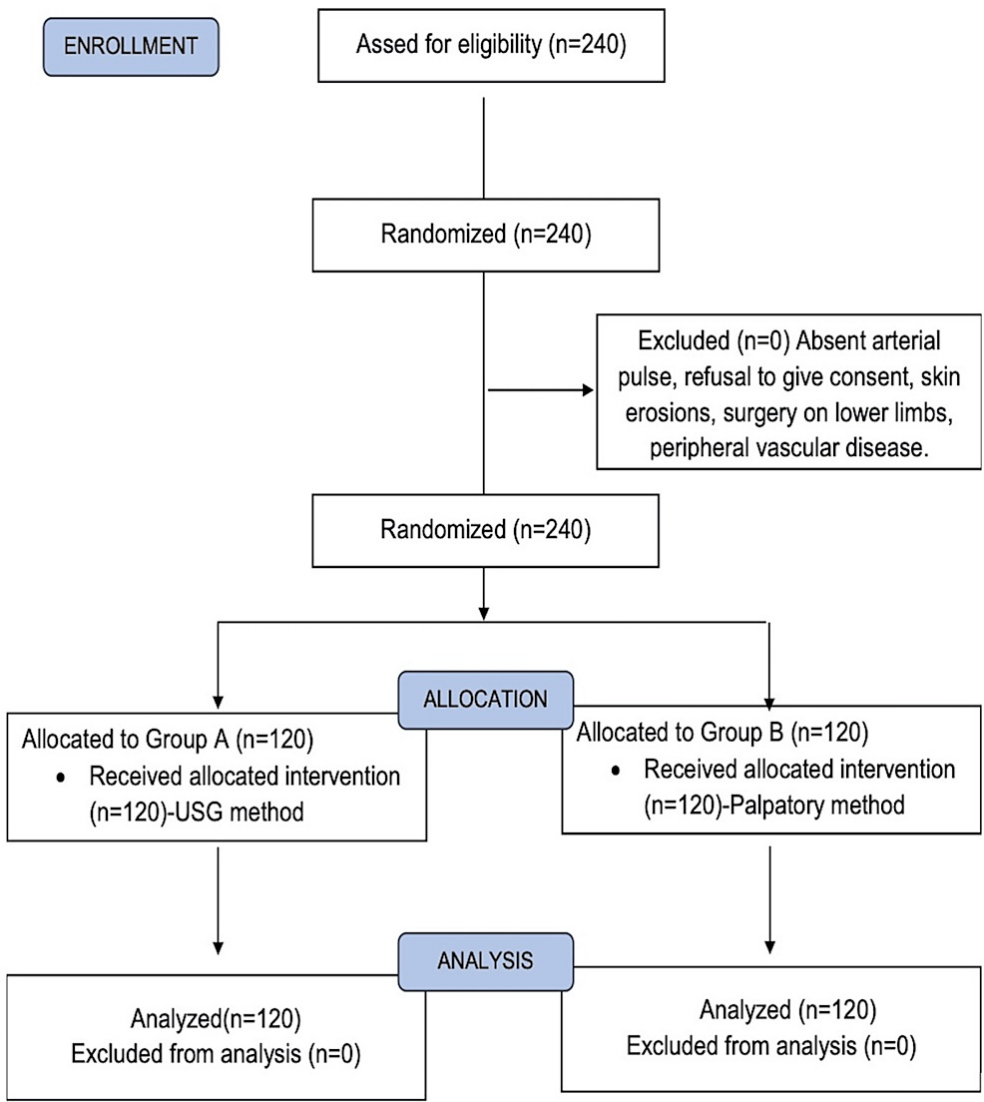
CONSORT flow diagram

A total of 240 patients were included and analyzed at the end of the study. Figure 1 shows the Consolidated Standards of Reporting Trials (CONSORT) flow diagram.

The baseline demographic characteristics of both groups were comparable in both the groups as depicted in Table 1.

**Table 1:** Demographic variables n = number, SD = standard deviation

Parameters	Groups		p-value
	Group A (n=120)	Group B (n = 120)	
Age (years), mean±SD	44.58 ± 15.82	41.33 ± 15.33	0.108
Gender			0.273
Male, n(%)	76 (63.3%)	84 (70.0%)	
Female, n(%)	44 (36.7%)	36 (30.0%)	
Weight (Kg), Mean±SD	59.17 ± 8.17	56.73 ± 5.87	0.844
ASA grade, n(%)			0.121
I	15 (12.5%)	18 (15.0%)	
IE	11 (9.2%)	11 (9.2%)	
II	45 (37.5%)	39 (32.5%)	
IIE	16 (13.3%)	23 (19.2%)	
III	12 (10.0%)	12 (10.0%)	
IIIE	12 (10.0%)	5 (4.2%)	
IV	0 (0.0%)	6 (5.0%)	
IVE	9 (7.5%)	6 (5.0%)	
Duration of surgery (hours), mean±SD	6.68 ± 2.68	6.71 ± 2.65	0.737
Heart rate (bpm), mean±SD	83.87 ± 8.97	82.88 ± 9.64	0.233
Systolic BP (mmHg), mean±SD	122.14 ± 13.64	121.82 ± 12.66	0.875
Diastolic BP (mmHg), mean±SD	77.84 ± 8.68	77.24 ± 8.91	0.482
SpO_2_ (%), mean±SD	99.15 ± 0.90	98.94 ± 0.96	0.090

Total successful cannulation in the first attempt among the study patients (n = 240) was seen in 19.2% (n = 46). Group A had 25.8% (n = 31) successful cannulation on the first attempt, and Group B had 12.5% (n = 15) successful cannulation on the first attempt (p = 0.009). The assessment time in Group A was 50.58 ± 9.29 seconds, and in Group B, it was 49.31 ± 9.35 seconds (Wilcoxon/Mann Whitney U test, p = 0.348). The mean cannulation time of Group A was 19.58 ± 5.11 seconds, and that of Group B was 19.41 ± 5.36 seconds (Wilcoxon/Mann-Whitney U test, p = 0.864), as depicted in Table 2.

**Table 2:** Comparison of cannulation characteristics in the two groups n = number

Parameters	Groups		p-value
	Group A (n=120)	Group B (n=120	
Successful cannulation in the first attempt, n(%)	31 (25.8%)	15 (12.5%)	0.009
Assessment time (seconds)	50.58 ± 9.29	49.31 ± 9.35	0.348
Time to cannulation (Seconds)	19.58 ± 5.11	19.41 ± 5.36	0.864
Number of attempts , n(%)			0.016
1	31 (25.8%)	15 (12.5%)	
2	38 (31.7%)	34 (28.3%)	
3	26 (21.7%)	29 (24.2%)	
>3	25 (20.8%)	42 (35.0%)	
Cannulation outcome			0.022
Success, n(%)	94 (78.3%)	78 (65.0%)	
Failure, n(%)	26 (21.7%)	42 (35.0%)	
Complications, n(%)			1.000
Nil	118 (98.3%)	117 (97.5%)	
Spasm	2 (1.7%)	3 (2.5%)	
Untoward events (%)			0.498
None	118 (98.3%)	120 (100.0%)	
No back flow after fixation	1 (0.8%)	0 (0.0%)	
No back flow after positioning	1 (0.8%)	0 (0.0%)	

The number of attempts for cannulation was one for 25.8% of the patients (n = 31), two for 31.7% of the patients (n = 38), three for 21.7% of the patients (n = 26), and more than three attempts for 20.8% (n = 25) of the patients in Group A, whereas it was one attempt for 12.5% of the patients (n =15), two for 28.3% of the patients (n = 34), three for 24.2% of the patients (n = 29), and more than three attempts for 35.0% of the patients (n = 42) in Group B (p = 0.016), as depicted in Table 2. In Group A, 78.3% of the patients (n = 94) had successful cannulation, with 21.7% (n = 26) having failure as an outcome. In Group B, 65% of the patients (n = 78) had successful cannulation, with 35% (n = 42) having failure as an outcome (p = 0.022), as depicted in Table 2. In Group A, two patients developed arterial spasms, and in Group B, three patients developed arterial spasms (p = 1.000).

## Discussion

Arterial cannulation is a commonly performed procedure in acute and critical care settings. It allows the monitoring of blood pressure with greater accuracy. Various superficial and easy-to-palpate arteries are commonly cannulated to measure arterial blood pressure. The main blood supply of the foot comes from three arteries, namely, the peroneal, anterior tibial, and posterior tibial arteries [6]. The PTA is a continuation of the popliteal artery. It is a deeply seated artery compared to the dorsalis pedis and radial artery [6]. It is palpated in the groove between the medial malleolus and calcaneus.

Sometimes, technical difficulties are encountered during arterial cannulation due to the small caliber of the artery, anatomical variation, arteriosclerosis, or vasospasm. Multiple attempts for cannulation are associated with a higher failure rate. Over the past decade, point-of-care US has been utilized routinely in operating rooms [7]. Arterial cannulation, under sonographic guidance, reduces the frequency of failed cannulation and cannulation-related complications [8]. It is even more useful when peripheral pulses are difficult to palpate, such as when a patient is in shock or extracorporeal membrane oxygenator [9]. 

In our study, the rate of successful first-pass PTA cannulation was higher in the USG group. The USG technique decreased the number of attempts required for arterial cannulation and increased the overall success rate. The mean assessment time, mean cannulation time, and rate of complications were similar between the two groups.

There have been mixed results on the utility of US for the cannulation of the radial artery. A meta-analysis by Gu et al. concluded that 2D US decreased the first attempt failure, mean attempts to success, and mean time to success of radial artery catheterization [10].

A study by Oulego-Erroz et al. on 128 critically ill pediatric patients concluded that US did not improve radial and femoral artery cannulation outcomes when done by experienced operators [11]. Another study by Yu et al. on radial arteries also concluded that the USG technique increased the first-attempt success rate with no significant difference in cannulation time. However, overall successful cannulation was not significantly different between the US and palpatory groups [12].

Similarly, Peters et al. compared palpatory and USG techniques for radial artery canulation in cardiac surgical patients and found no significant difference between the both groups [13]. It is crucial that the first attempt of cannulation becomes successful since failed cannulation attempts and multiple attempts will lead to arterial vasospasm, hematoma formation, nearby nerve damage, and arterial thrombosis [14]. In our study, the first attempt at successful cannulation was greater in the US group when compared to the palpatory method, although the median time required for successful cannulation was not significantly different in both groups. This was also supported by a study by Eun-hee et al., who stated the benefits of the PTA as an arterial cannulation site and a reasonable alternative to the radial artery for USG arterial cannulation in children [4]. Very recently, Takeshita et al. compared USG arterial catheterization of the radial, dorsalis pedis, and PTA in children of <3 years of age and concluded that USG cannulation of the dorsalis pedis and PTA was not inferior to USG radial artery cannulation [15].

Anand et al. compared the US technique with the palpatory technique for the dorsalis pedis artery and found no difference with respect to first-attempt success rate and the number of cannulation attempts [3]. As the PTA is relatively deeply located, the US technique might lead to a significantly higher success rate than more superficial arteries, such as the dorsalis pedis and radial artery. The anatomical location of the PTA also helps in placing and stabilizing the US probe.

With increasing attempts, there is a risk of arterial vasospasm and hematoma formation. We also found a significantly lower number of attempts required for successful cannulation in the USG group. USG gives real-time imaging of the artery. It allows measurement of the depth of the artery from the skin, the diameter of the artery, the course of the artery, and visualization of nearby structures. These assessments are not possible with the palpatory technique. One recent study also compared the PTA cannulation with USG and the palpation technique. The authors found that the successful PTA cannulation rate on the first attempt was comparable between the USG and palpation groups. By contrast, the cannulation time and total procedural time were significantly higher in the palpation group [16].

The difference in the findings in this study may be explained by the fact that a much higher number of patients were enrolled in our study (n = 240) compared to theirs (n = 76). Our patients were of wide range of ASA class (I-IV).

Our study had certain limitations. Although a single investigator performed the PTA calculations, he was far more experienced in palpation technique for the same. Moreover, the results of this study cannot be generalized to all children.

## Conclusions

This study aimed to find the preferred technique (palpatory versus USG) for PTA cannulation. The present study demonstrates that the USG technique for PTA cannulation may be preferred as the first attempt of successful cannulation, and overall successful cannulation was greater and statistically significant in the USG group than in the palpatory group. However, the assessment time and cannulation time are comparable in both groups.
